# Transcatheter Edge-to-Edge Repair of Mitral Valve Regurgitation: Closing the Gap to Broaden the Coverage

**DOI:** 10.31083/j.rcm2401015

**Published:** 2023-01-06

**Authors:** Hafiz Imran, Khansa Ahmad, Muhammad Baig, Islam Y. Elgendy, Nasir Iqbal, Afshin Ehsan, Barry Sharaf, Paul Gordon, Marwan Saad

**Affiliations:** ^1^Department of Medicine, Division of Cardiology, Alpert Medical School of Brown University, Providence, RI 02903, USA; ^2^Lifespan Cardiovascular Institute, Providence, RI 02903, USA; ^3^Division of Cardiovascular Medicine, Gill Heart Institute, University of Kentucky, Lexington, KY 40536, USA; ^4^Division of Cardiovascular Medicine, Azra Naheed Medical College, 55150 Lahore, Pakistan; ^5^Division of Cardiothoracic Surgery, The Warren Alpert Medical School of Brown University, Providence, RI 02903, USA

**Keywords:** mitral regurgitation, transcatheter edge-to-edge repair of mitral valve regurgitation, acute ischemic mitral regurgitation, atrial mitral regurgitation

## Abstract

**Background:**

Transcatheter edge-to-edge repair of mitral valve (M-TEER) is reasonable consideration in symptomatic patients with severe degenerative mitral regurgitation (MR) who are at high or prohibitive risk of surgical repair or replacement. In symptomatic patients on maximally tolerated medical therapy with severe secondary MR from left ventricular systolic dysfunction, M-TEER is reasonable therapeutic option.

**Methods:**

In this review, we present a comprehensive overview of the most recent literature and considerations for M-TEER in patients excluded from key trials. These include patients with cardiogenic shock, acute ischemic MR, atrial functional MR, failed surgical mitral valve prosthesis and pulmonary hypertension.

**Conclusions:**

M-TEER is feasible and a reasonable alternative option for these patient populations with a significant clinical benefit. However, randomized clinical trials are needed to ascertain findings from these observational studies.

## 1. Background

Mitral regurgitation (MR) can occur either due to primary degenerative pathology of the mitral valve (degenerative MR) or secondary to other cardiac pathology (functional MR) such as annular dilation, ventricular dilation (e.g., dilated cardiomyopathy), atrial dilation (e.g., atrial fibrillation), or papillary muscle dysfunction (e.g., coronary artery disease). If untreated, significant MR leads to progressive left ventricular systolic dysfunction which results in higher than 5% annual mortality among symptomatic individuals [1]. While medical management can help with symptom relief, it is unlikely to halt the progression of the underlying pathology. Open surgical repair remains the definitive treatment for degenerative mitral valve disease. However, a significant number of patients do not undergo surgery due to profound left ventricular systolic dysfunction, advanced age, or multiple co-morbidities which increase the risk of peri-operative morbidity and mortality [2]. Transcatheter edge-to-edge repair (M-TEER) with the ${\mathrm{MitraClip}}^{\text{TM}}$ is an alternative to surgery in patients at high risk for surgical valve repair or replacement. This review aims to examine the current role of M-TEER in treating patients with MR and highlight the challenges with this therapy in the subgroups of patients who were excluded from the landmark randomized controlled trials (RCTs).

## 2. Invention and Approval of M-TEER

M-TEER was developed based on the concept of surgical Alfieri stitch. This surgical procedure aims to reduce MR by sewing together the anterior and posterior leaflets where the valve is incompetent. The patent for the ${\mathrm{MitraClip}}^{\text{TM}}$ device (Abbott Laboratories, Chicago, IL, USA) was filed in 1997 and the first case was performed in Venezuela in 2003. The device is a fabric-covered nitinol clip with 2 arms and associated grippers mounted at the end of a delivery system. The procedure is performed with fluoroscopic and transesophageal echocardiographic guidance under general anesthesia. The clip is advanced into the left atrium through a trans-septal puncture and after adjusting its orientation towards the area of mitral pathology or maximum regurgitation, the device is advanced into the left ventricular cavity. The anterior and posterior mitral leaflets are grasped at the point of maximum regurgitation and the ${\mathrm{MitraClip}}^{\text{TM}}$ is released after confirming adequate tissue grasping and a significant reduction in mitral regurgitation, without a significant increase in the transvalvular gradient. More than one clip may be required to reduce the degree of mitral regurgitation by 2 grades or more.

The first RCT to examine the role of M-TEER was the Endovascular Valve Edge-to-Edge Repair Trial (EVEREST II). A total of 279 patients eligible for mitral valve repair or replacement with chronic 3+ or 4+ degenerative MR were randomly assigned to M-TEER or surgical mitral valve repair or replacement in a 2:1 fashion. Amongst participants, symptomatic patients had left ventricular ejection fraction of >25% and left ventricular end-systolic volume of 55 mL or less. Asymptomatic patients had one of the following: left ventricular ejection fraction of 25–60%, left ventricular end-systolic diameter 40 to 55 mm, new-onset atrial fibrillation, or pulmonary hypertension. Anatomic inclusion criteria required the origin of primary regurgitant jet to be from malcoaptation of the middle scallops of the anterior and posterior leaflets. M-TEER achieved similar clinical outcomes (composite of freedom from death, surgery for mitral valve dysfunction, and grade 3+ or 4+ mitral regurgitation) at 12 months with better safety outcomes when compared with surgical repair [3]. At 5-year follow-up, mortality in as-treated population was similar between M-TEER and surgical repair or replacement, leading to the approval of ${\mathrm{MitraClip}}^{\text{TM}}$ for M-TEER by the Food and Drug Administration (FDA) in 2013 for patients with prohibitive surgical risk and ≥3+ degenerative mitral regurgitation [4]. The recent ACC/AHA guidelines recommend M-TEER as a reasonable alternative to surgical repair in severely symptomatic patients (NYHA class III and IV) with severe degenerative MR who are at high or prohibitive risk, if anatomy is favorable and life expectancy is at least one year [5].

In patients with functional MR, the role of M-TEER was evaluated in 2 RCTs with conflicting results. Percutaneous Repair with the ${\mathrm{MitraClip}}^{\text{TM}}$ Device for Severe Functional/Secondary Mitral Regurgitation (MITRA-FR), conducted in Europe, included 279 patients with heart failure (HF) and severe secondary MR on guideline-directed medical and cardiac resynchronization therapies randomized to M-TEER vs. conservative therapy [6]. There was no difference between M-TEER and medical therapy alone for the composite of death and HF hospitalization at 12 months. In contrary, The Cardiovascular Outcomes Assessment of the ${\mathrm{MitraClip}}^{\text{TM}}$ Percutaneous Therapy for Heart Failure Patients with Functional Mitral Regurgitation (COAPT) trial, conducted in the United States and Canada randomized 610 patients with HF and moderate-to-severe or severe secondary MR with NYHA class II-IV symptoms on optimal medical and cardiac resynchronization therapies to M-TEER vs. ongoing medical therapy [7]. Contrary to MITRA-FR, the COAPT trial demonstrated a significant reduction in HF hospitalization and mortality in patients that underwent M-TEER compared to medical therapy alone.

The discrepancy in outcomes between the two trials was attributed to the difference in characteristics of respective cohorts. In the MITRA-FR trial, participants had a higher degree of left ventricular dilatation and more “proportionate” degree of MR compared to the COAPT trial which had more patients with “disproportionate MR”, i.e., effective orifice area of 0.3 to 0.4 ${\mathrm{cm}}^{2}$ but with left ventricular end-diastolic volume of only 160 to 200 mL. When compared to patients in MITRA-FR trial, COAPT trial patients had a 30% higher effective regurgitant orifice with a 30% lower left ventricular end-diastolic volume, i.e., disproportionate MR was the predominant phenotype [8]. Additionally, in the COAPT trial, more patients were on maximally tolerated guideline-directed medical therapy and had received appropriate device therapy or revascularization prior to randomization. Furthermore, very few adjustments in medical therapy were made during the follow-up period. On the other hand, in the MITRA-FR trial, medical therapy was not optimized in all patients at baseline and multiple adjustments were made during the follow-up period in both arms. These major differences in the selection of trial participants and optimization of medical therapy likely explain the discrepancy in clinical outcomes between the 2 trials. The COAPT trial led to the FDA approval of M-TEER for functional severe mitral regurgitation in 2019. The recent ACC/AHA guidelines recommend M-TEER as a reasonable therapy in patients with severe functional MR related to left ventricular systolic dysfunction (LVEF <50%) with persistent symptoms (NYHA class II, III, or IV) on optimal medical therapy for HF and appropriate anatomy defined by transesophageal echocardiogram and left ventricular systolic function 20–50%, left ventricular end-systolic dimensions ≤70 mm, and pulmonary artery systolic pressure ≤70 mmHg [5].

Initial real-world data from the STS/TVT registry on 564 patients who underwent M-TEER showed promising results. The predicted 30-day mortality for either surgical mitral valve repair or replacement was 7.9% and 10.0% respectively, for patients treated with M-TEER. Most of these patients had degenerative mitral valve disease, and M-TEER was successful (defined as final MR grade ≤2) in 93% of patients with a 30-day mortality of 5.8% [9].

Even though technical success for this procedure is high, appropriate selection of patients is of paramount importance to achieve clinical benefits. Over the last decade, there has been a significant increase in the number of M-TEER procedures with improved clinical outcomes even in relatively complex patient populations [10]. This significant increase in M-TEER volume has been driven by patients with degenerative MR [11]. While the landmark trials have included patients with both degenerative and functional MR, M-TEER has also been successfully performed in patients who otherwise would be excluded from these trials.

## 3. Current Challenges and Future Directions

### 3.1 ${\mathrm{MitraClip}}^{\text{TM}}$ in Special Populations

#### 3.1.1 Patients with Cardiogenic Shock

In trials of M-TEER, patients with cardiogenic shock were excluded. However, recent reports support the feasibility and potential benefit of the therapy in this complex patient population. Most recently, the largest data on the efficacy of M-TEER in patients with severe MR and cardiogenic shock (CS) was presented at TCT as a late-breaking study from STS/TVT/ACC registry [12]. CS was defined by hypotension and severe reduction in cardiac index (<1.8 L/min/$m^{2}$ without support or <2.2 L/min/$m^{2}$ with support) with end-organ hypoperfusion. A total of 3,797 patients were identified with a mean age of 73.0 ± 11.9 years. The etiology of MR was degenerative, functional, and mixed in 53.4%, 27.5%, and 16.3% of patients, respectively. Device success was achieved in 85.6% of patients. Final MR ≤2 was achieved in 88.2% of the patient population. At 1-year, device success was associated with reduced all-cause mortality (34.6% vs. 55.5% adjusted-HR 0.49, 95% confidence interval [CI] 0.41–0.59, *p *< 0.001), and composite of mortality and HF hospitalization (29.6% vs. 45.2%, adjusted HR 0.51, 95% CI 0.42–0.62, *p *< 0.001).

A multicenter registry data of acute myocardial infarction (AMI) patients was used to examine the impact of M-TEER in patients with and without CS during index hospitalization [13]. A total of 93 patients were included, 50 of whom were diagnosed with CS. Technical success was similar between groups (90% vs. 93%, *p* = 0.79) with no significant difference in all-cause mortality at 30-days (10% vs. 2.3%, *p* = 0.21) and 7-months (16% vs. 9.3%, *p* = 0.38). There was also no difference in combined death/hospitalization due to heart failure at 7 months (28% vs. 25.6%, *p* = 0.79). M-TEER was performed approximately 24 days after AMI in patients with CS with the goal to achieve hemodynamic stability from shock before consideration for M-TEER. These studies suggest that M-TEER can serve as a safe and effective alternative for treating MR in patients who are not deemed to be candidates for open surgical repair or replacement.

A patient-level multicenter analysis of patients with CS (n = 141) and moderate to severe or severe mitral regurgitation further supported the feasibility of M-TEER in this patient population [14]. The majority of patients were in Society for Cardiovascular Angiography and Interventions (SCAI) shock stage C (50.4%) or D (29.8%) and half of the patients were on mechanical circulatory support. Procedural success was achieved in 88.7% of the patients. In-hospital, 90-day, and 1-year mortality occurred in 15.6%, 29.5%, and 42.6%, respectively. In patients who had procedural success, M-TEER reduced in-hospital (hazard ratio [HR] 0.36; 95% CI 0.13 to 0.98; *p* = 0.04) and 90-day (HR 0.36; 95% CI 0.13 to 0.78, *p* = 0.01) mortality, and the composite of 90-day mortality and HF hospitalization (HR 0.41; 95% CI 0.19 to 0.90, *p* = 0.03) compared to patients in whom procedural success could not be achieved [14].

MITRA-SHOCK is a retrospective multicenter study that reported the outcomes of M-TEER in 31 patients with refractory CS treated with inotropes and diuretics with or without mechanical circulatory support. These patients were deemed inoperable by a heart team per site protocol, with an STS risk score for mitral valve surgical repair of 37.9 (Inter Quartile Range 30.4–42.4). M-TEER was pursued for compassionate care without any specific study protocol. Among the 31 patients, 24 had dilated cardiomyopathy and 17 had STEMI. Procedural success was achieved in 87% of patients with significantly higher survival in patients with procedural success when compared with those without procedural success (87.2%; 95% CI 73–99% vs. 25%; 95% CI 4.6–96%, *p *< 0.001) [15].

Data from these observational studies suggest reasonably high procedural success in this critically ill population with CS with significant improvement in short- and mid-term outcomes. The Transcatheter Mitral Valve Repair for Inotrope Dependent Cardiogenic Shock (CAPITAL-MINOS) trial is enrolling patients to assess the efficacy of M-TEER in patients with inotrope-dependent cardiogenic shock (SCAI Stage C & D) with at least 3+ MR [16]. The study will evaluate a composite endpoint of in-hospital all-cause mortality, cardiac transplantation, left ventricular assist device implantation, or discharge on palliative inotropes. Results from this trial would further guide our decision-making in this select patient population.

#### 3.1.2 Patients with Acute Ischemic MR

Acute severe MR after myocardial infarction in patients treated with primary percutaneous intervention is associated with poor clinical outcomes when compared with patients who do not develop mitral regurgitation [17, 18]. Emergent surgical repair or replacement remains the gold standard therapy, however, most of these patients are at high or prohibitive risk for surgical intervention due to acute myocardial infarction and hemodynamic instability [19]. Those treated medically have the worst outcomes [20]. Pharmacologic afterload reduction and mechanical circulatory support are supportive options until definitive treatment can be provided. The availability of M-TEER offers an additional treatment option, but the landmark trials (MITRA-FR and COAPT) excluded this subset of patients.

Single- and multi-center case series have reported successful reduction of MR with M-TEER, which translated into short-term clinical benefits [21, 22]. The International Registry of ${\mathrm{MitraClip}}^{\text{TM}}$ in Acute Mitral Regurgitation following Acute Myocardial Infarction (IREMMI) was created to assess clinical outcomes in patients who underwent M-TEER for moderate to severe or severe ischemic MR after AMI. A recent analysis from the IREMMI registry showed promising results after mitral valve intervention [23]. Amongst 471 patients included in the analysis, 205 underwent mitral valve intervention (surgical n = 106 vs. M-TEER n = 99). Patients undergoing mitral intervention had lower in-hospital and 1-year mortality when compared with patients who were medically managed (11% vs. 27%, *p *< 0.01 and 16% vs. 35%, *p *< 0.01; adjusted HR 0.28; 95% CI 0.18 to 0.46, *p *< 0.01). Surgical mitral intervention was performed sooner than M-TEER (median 12 days in surgical arm vs. 19 days in M-TEER arm) after AMI. Technical success was similar between surgical and M-TEER groups (92% vs. 93%). Inpatient and 1-year mortality were significantly lower in M-TEER group (6% vs. 16%, *p* = 0.03 and 17% vs. 31%, *p* = 0.04, adjusted HR 3.75 and 95% CI 1.55 to 9.07, *p *< 0.01) compared to the surgical group. A significant proportion of patients experienced cardiogenic shock (surgical 31% vs. M-TEER 52%). This multicenter data suggests that M-TEER offers similar technical success rates with improved mortality when compared with surgical intervention in patients with acute post-MI severe MR, despite a sicker population treated with M-TEER. Of note, this analysis excluded patients who developed acute severe MR due to a ruptured papillary muscle following AMI, since M-TEER is not a reliable option in this population. Additionally, 1-year survival was similar among discharged patients who underwent M-TEER or surgical mitral valve repair during index hospitalization. The findings support the role of M-TEER as a valid treatment option in acute severe ischemic mitral MR. A randomized trial would provide further evidence to support the use of M-TEER vs. corrective mitral valve surgery and medical management in this complex and high-risk patient population.

#### 3.1.3 Patients with Functional MR due to Atrial Dilation

Functional MR from atrial dilation (e.g., in patients with atrial fibrillation) is secondary to annular dilatation which differs from functional MR from ventricular dilatation in patients with HF. In the prospective, observational, multicenter EXPAND (A Contemporary, Prospective, Multi-Center Study Evaluating Real-World Experience of Performance and Safety for the Next Generation of MitraClip Devices) study patients with atrial functional MR were identified by an echocardiography core laboratory. Device success was achieved in 100% patients at 1-year with significant improvement in functional class and Kansas City Cardiomyopathy Questionnaire score. These results were similar to patients with functional MR of ventricular dysfunction [24]. A recent study of 1044 patients compared 2-year outcomes in patients with degenerative (48%), atrial functional (11%), and ventricular functional (48%) MR with a mean STS Score of 8.6 ± 7.8 who underwent M-TEER. A composite of all-cause mortality and HF hospitalization was significantly higher in atrial (31.5%) and ventricular (42.3%) functional MR when compared with degenerative MR (21.6%) even with successful M-TEER (log-rank *p *< 0.001) [25]. A single-center experience from Germany reported technical success of 94.1% and MR reduction to ≤1 (79.7%) among 118 patients with atrial functional MR. The use of newer generation ${\mathrm{MitraClip}}^{\text{TM}}$ systems (NTR/XTR or G4 systems) was associated with higher rates of MR reduction to ≤1 [26]. A single arm data from the Italian MITRA-TUNE registry reported outcomes in atrial functional MR patients at a follow-up period of 2 years [27]. Procedural success was 80% at 30-days with all-cause mortality of 5%. Freedom from all-cause mortality at 2 years was 60% and the composite of freedom from all-cause mortality and HF hospitalization was 55%. M-TEER was associated with positive remodeling of left atrial and mitral annular sizes. Improvement to NYHA functional class I/II was achieved in 79% patients at a median follow-up of 455 (IQR 234–1013) days.

Recently, a few studies from Europe have reported outcomes after M-TEER in patients with atrial functional severe MR and compared outcomes with ventricular functional severe MR [28, 29]. There was improvement in NYHA functional class ≤ II at 6 months follow-up in atrial (90%) vs. ventricular (80%) was similar (*p* = 0.2) [28]. Registry data from Spain including 48 patients who underwent M-TEER for atrial functional MR showed a procedural success of 91.7% that lasted to the study’s 12-month follow-up. There was a significant improvement in New York Heart Association (NYHA) functional class at 6 and 12 months (Baseline: NYHA III 70.8%, NYHA IV 18.8% vs. 1-year: NYHA III 21.7%, NYHA IV 0%, *p *< 0.001). Freedom from HF hospitalization and all-cause mortality was 74.9% which was comparable to outcomes after M-TEER for ventricular functional severe MR [29].

Similarly, a Belgian registry reported outcomes of 52 patients with atrial functional MR compared with ventricular functional MR (n = 307) [28]. Reduction in MR was greater when compared with ventricular functional MR (94% vs. 82%, *p *< 0.001) at 6 months. Additionally, cardiac output at rest (5.1 ± 1.5 L/min vs. 3.8 ± 1.5 L/min, *p* = 0.002) and exercise (7.9 ± 2.4 L/min vs. 6.1 ± 2.1 L/min, *p* = 0.02) was significantly higher in atrial MR vs. ventricular MR. Reduction in pulmonary arterial systolic pressure (PASP) was higher in atrial functional MR patients than in ventricular functional MR patients (Δ PASP –13.1 ± 15.1 mmHg vs. –2.2 ± 13.3 mmHg, *p* = 0.03). Clinical outcomes, driven by HF hospitalization, were also lower in the atrial functional MR group than in the ventricular functional MR group (adjusted odds ratio of 0.46, 95% CI 0.24 to 0.88). Data from these two retrospective studies suggest that even though the mechanism of functional MR of atrial origin is different, these patients still derive clinical, anatomic, and hemodynamic benefits from M-TEER. We think that apparently better clinical outcomes of m-TEER in atrial functional MR than ventricular functional MR may be due to multiple reasons. First, ventricular dysfunction independently increases mortality. M-TEER does not affect primary pathophysiologic mechanism of ventricular dysfunction rather decreases secondary effects from congestion related to dysfunction. Second, in patients with atrial functional MR, mitral regurgitation potentially further worsens atrial dilatation and when MR is corrected by M-TEER, it slows down associated atrial dilatation.

#### 3.1.4 Patients with Failed Surgical Mitral Repair

Surgical mitral valve repair is the preferred treatment for degenerative severe MR in patients at acceptable surgical risk. Patients with prior surgical mitral repair were excluded from all RCTs due to the change in the anatomy of the mitral apparatus and concerns about the technical feasibility to achieve success with M-TEER. The presence of a mitral ring can obscure posterior leaflet visualization during the grasp and limit orifice dimensions for passage of the ${\mathrm{MitraClip}}^{\text{TM}}$ from the left atrium to the left ventricle in the recommended configuration. A multicenter retrospective analysis of data shows comparable technical and device success for M-TEER at 90% and 89% amongst 104 consecutive patients who had previously undergone surgical mitral valve repair. Residual MR was moderate or less in 90% of the patients [30]. In-hospital mortality was 2%, and 86% of patients were in NYHA class ≤II at 6-month follow-up. Additional or modified imaging was applied in 21% of cases to overcome the limitations of the change in anatomy, including the use of intracardiac echocardiography, or TEE with trans-gastric short-axis views or left lateral position in mid-esophageal views. Despite limited data in the literature, it appears that with some modifications in technique and imaging modality/views, M-TEER represents a feasible option in selected patients at higher risk for redo-surgical intervention. Similarly, edge-to-ring has been attempted in some cases to achieve reduction in MR in failed mitral surgical repair [31].

#### 3.1.5 Patients with Pulmonary Hypertension

Development of pulmonary hypertension (PH) in patients with severe mitral regurgitation is an indication for mitral valve repair or replacement [5]. Worse clinical outcomes have been reported in patients with PH who undergo surgical mitral valve repair or replacement [32]. Different studies have reported effect of M-TEER on PH and effect of pre-existing PH on clinical outcomes after M-TEER. In an initial study of 91 patients with functional MR, procedural success and 30-day mortality were similar in PH group (pulmonary artery systolic pressure (PASP) ≥50 mmHg by echocardiogram) and non-PH group [33]. There was significant improvement in PASP from baseline, but PASP remained higher than non-PH group. At a mean follow up of 25.0 ± 16.9 months, there was significantly higher all-cause mortality in PH group (HR 3.73, 95% CI 1.65–8.47, *p* = 0.002). An analysis from German Transcatheter Mitral Valve Intervention (TRAMI) registry divided patients into 3 groups based on PASP by echocardiogram, i.e., group I: PASP ≤36 mmHg, group II PASP 37–50 mmHg, and group III: PASP >50 mmHg. Procedural success, in-patient and 30-day mortality were similar across 3 groups [34]. However, a composite of 1-year all-cause mortality and major cardiovascular events was higher in group 2 (33.1%) and group 3 (34.7%) than group 1 (20.3%, *p *< 0.01). Both groups had higher predictive mortality (group 2; HR 1.81, *p* = 0.01 and group 3; HR 1.85, *p *< 0.01). Largest analysis of patients (n = 4071) from STS/ACC registry divided patients into 4 groups based on invasive mean pulmonary artery pressure (mPAP): Group 1 with no PH (mPAP <25 mmHg), group 2 with mild PH (mPAP 25–34 mmHg), group 3 with moderate PH (mPAP 35–44 mmHg), and group 4 with severe PH (mPAP ≥45 mmHg) [35]. A composite of 1-year mortality and HF hospitalization was higher in group 2 (32.4%, 95% CI, 29.0–35.8%), group 3 (36.0%, 95% CI, 31.8–40.2%) and group 4 (45.2%, 95% CI, 39.1–51.0%) than group 1 (27.8%, 95% CI 24.2–31.5%). After multivariable adjustment, PH was associated with higher 1-year mortality (HR per 5 mmHg mPAP increase, 10.5; 95% CI 1.01–1.09; *p* = 0.02). Procedure was unsuccessful in overall 3.1% patient. There was significant improvement in NYHA functional class across all groups, however PH was associated with persistent higher NYHA functional class III (group 1; 9.5% vs. group 4; 13.3%, *p *< 0.01) and class IV (group 1; 1.2% vs. group IV; 4.2%, *p *< 0.01). Kottenberg and colleagues reported immediate effect of M-TEER on mPAP amongst patients undergoing procedure under general anesthesia. There was 10% decrease in mPAP irrespective of pre-procedure diagnosis of PH (mPAP >25 mmHg) [36]. However, Mandurino-Mirizzi and colleagues studied impact of M-TEER on reduction of mPAP 6-months post-procedure without sedation (excluding any effect of anesthetic medications). Reduction in mPAP was numerically similar, though statistically nonsignificant, to prior study by Kottenberg and colleagues [37]. Concept of vasoreactivity testing to sodium nitroprusside in patients with severe MR and post-capillary PH (mPAP >20 mmHg and pulmonary artery wedge pressure (PAWP) >15 mmHg) in 22 patients was introduced to identify patients who would get most benefit from procedure [38]. A positive test (responders) was defined by normalization of mPAP and reduction in PAWP to ≤15 mmHg with incremental doses of sodium nitroprusside. Patients underwent M-TEER after initial right heart catheterization. At 6-months, a follow-up right heart catheterization showed significant improvement in cardiac index (+0.45, 95% CI: +0.61 to +0.29 L/min/$m^{2}$, *p* = 0.001). However, mPAP significantly improved from 39 mmHg (range 37–42) to 28 mmHg (range 23–31) amongst responders when compared with non-responders (baseline; 40 mmHg with range 36–41 and 6-months; 41 mmHg with range 40–43) with *p *< 0.001. However, this unique concept needs testing in a larger population and definition of relationship between vasoreactivity responsiveness and clinical outcomes.

### 3.2 Impact of Mitral Gradient after M-TEER?

Among the concerns of surgical mitral valve repair is the impact of reduced mitral valve area and elevated gradient on clinical outcomes after repair. This is more of a concern with M-TEER compared with surgery as many cases require more than 1 clip to achieve procedural success and clinical benefit, especially in those with small pre-procedural valve area (<4 ${\mathrm{cm}}^{2}$). Initial data showed that elevated post-procedural mitral valve gradients (>4.4 mmHg) across the mitral valve were associated with worse clinical outcomes in patients with severe MR and HF [39]. However, a more recent report of M-TEER in 255 patients showed no difference in clinical outcomes between post-procedural mitral valve gradient of more vs. less than 4.4 mmHg at a median follow-up period of ~19 months [40]. In a subgroup analysis based on the type of MR (primary vs. secondary), post-procedural mitral valve gradient >4.4 mmHg predicted a worse clinical outcome (a combined endpoint of all-cause mortality, mitral valve surgery, redo procedure, implantation of left ventricular assist device [*p* = 0.03]) in patients with degenerative MR. In contrast, patients with functional mitral regurgitation did not have any difference in clinical outcomes if post-procedural mitral valve gradients were lower vs. higher than 4.4 mmHg (n = 20). In the same study, the authors analyzed the clinical outcomes based on residual MR. Moderate or more residual MR was associated with worse clinical outcomes when compared with patients who had mild or minimal residual MR. A supplementary analysis showed less favorable clinical outcomes associated with the combination of residual moderate MR and post-procedural mitral valve gradient ≤4.4 mmHg than for minimal or mild MR and a post-procedural mitral valve gradient >4.4 mmHg [40]. These results are consistent with prior data in patients with chronic ischemic mitral regurgitation who underwent surgical mitral valve repair with an undersized ring who developed mitral valve gradients >5 mmHg, but without difference in clinical outcomes [41].

A recent post-hoc analysis of patients in the COAPT trial based on post-procedural mitral valve gradients of 2.1, 3.0, 4.2, and 7.2 mmHg showed no difference in a composite clinical end point of all-cause mortality, HF hospitalization and health status at the end of 2-year follow-up [42]. Another recent study analyzed outcomes after M-TEER amongst patients with primary mitral regurgitation. There was no difference in the composite outcome of all-cause mortality and HF hospitalization in patients with a post-procedural mitral valve gradient of 6.0 mmHg vs. 1.9, 3.0, or 4.0 mmHg (n = 419) [43].

Although initial data was concerning for worse clinical outcomes if post-procedural mitral valve gradients were >4.4 mmHg for degenerative or functional MR, emerging data supports pursuing procedural success even if post-procedural gradients are up to even 7 mmHg, irrespective of MR etiology.

Even though, M-TEER has been performed in both degenerative and functional MR patients, significant challenges exist in certain populations. There is increased risk of mitral stenosis following M-TEER in patients with severe mitral annular calcification, calcified leaflets and multiple regurgitant jets. Adequate reduction in MR may not be achieved in patients with cleft mitral leaflet, short posterior leaflet (<5 mm), tethered leaflets and large coaptation gap. In addition, M-TEER may not be technically feasible in patients with prior catheter-based closure of atrial septal defects. Role of M-TEER to avoid progression of underlying HF as bridge-to-transplant, bridge-to-candidacy and bridge-to-decision making in patients with advanced HF was reviewed in a recent study. But results of randomized trial may guide us further about role of M-TEER in this group. Outcomes of M-TEER in elderly patients with degenerative MR and low-intermediate surgical risk were compared against isolated surgical mitral valve repair using a propensity score model in this retrospective cohort. Though a significant number of patients in M-TEER group had severe TR at baseline suggestive of advanced disease, survival at 1-year was similar in both groups. Randomized trial in low-intermediate risk group patients with newer generation devices may expand role of M-TEER in patients with severe degenerative MR.

## 4. Conclusions

**Fig. 1.**
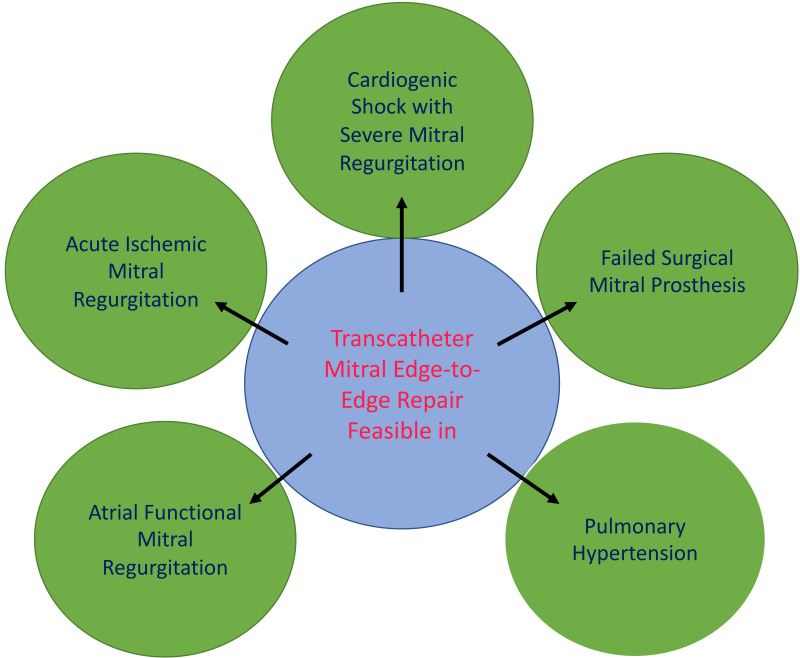
**Feasibility of transcatheter mitral edge-to-edge repair in different patient populations**.

Since the reporting of landmark trials, M-TEER has emerged as a reasonable alternative to surgical mitral valve intervention in both degenerative and functional MR. Even though these trials excluded critically and acutely ill patients, observational data from single- and multi-center registries are encouraging, especially when significant reduction of MR is achieved (see Fig. 1). Randomized controlled trials are needed to verify the promising results of these observational studies. Significant challenges still exist in patients with small valve area, calcified leaflets, multiple regurgitant jets, cleft leaflets, short posterior leaflet (length <5 mm), tethered leaflets, and large coaptation gaps. Future transcatheter mitral valve replacement devices are much awaited for patients who are not candidates for surgery or M-TEER.
